# Electric Conductivity Transitions of Water-Absorbable Polybenzimidazole Films

**DOI:** 10.3390/polym17020167

**Published:** 2025-01-11

**Authors:** Kaito Watanabe, Junko Ikeda, Xianzhu Zhong, Jiabei Zhou, Tatsuo Kaneko, Mika Kawai, Tetsu Mitsumata

**Affiliations:** 1 Graduate School of Science and Technology, Niigata University, Niigata 950-2181, Japan; 2 Mageleka Japan Co., Ltd., Kashiwa 277-0882, Japan; 3 Graduate School of Advanced Science and Technology, Japan Advanced Institute of Science and Technology, Nomi 923-1292, Japan; 4 School of Chemical and Material Engineering, Jiangnan University, Wuxi 214122, China

**Keywords:** heat-resistant polymer, polybenzimidazole, water absorption, electric resistivity, dielectric constant, time-domain NMR

## Abstract

Transitions seen in the electric properties of water-absorbable poly(2,5-benzimidazole) (ABPBI) films were confirmed by electric conductivity, dielectric constant, and time-domain nuclear magnetic resonance (NMR) measurements. The electric resistance of the films was measured at room temperature using a high-resistance meter, and the dielectric constant at room temperature was measured using an LCR meter in the frequency range of 90 Hz to 8 MHz. The water absorption ratio at equilibrium absorption for the films was 37%, which corresponded to a volume fraction of water of 0.33. The electric conductivity of the films without water absorption was ~10^14^ S·cm^−1^, and it increased to ~10^10^ S·cm^−1^ with increasing volume fraction, showing a percolation threshold at a volume fraction of 0.025, and remarkable transitions at volume fractions of 0.075 and 0.135. The dielectric constant of the films without water absorption was 3.4, and it increased to 8.1 with increasing volume fraction, showing a transition only at a volume fraction of 0.135. Above a volume fraction of 0.075, where a transition in conductivity was observed, there were two relaxation times at 18–31 μs and 20–93 μs, as determined from the time-domain NMR, and these relaxation times increased with increasing volume fraction. The longer relaxation time increased significantly at a volume fraction of 0.072, which was close to the volume fraction of the transition seen in conductivity. The relationship between the chain mobility of ABPBI and the deterioration in electric insulating properties is discussed.

## 1. Introduction

Heat-resistant polymers have been studied very actively for almost a century, and they are now widely used in a variety of industrial products. Recently, many papers on batteries [1,2], fuel cells [3,4,5], and ion-exchange membranes [6,7] have been published. As polymers are inherently lightweight, heat-resistant polymers can be used to replace inorganic materials such as metals and ceramics if the heat-resistance temperature can be increased. In recent years, where weight reduction is important, the advantage of heat-resistant polymers is very significant. In particular, poly(2,5-benzimidazole) (ABPBI), which can be prepared using a bio-based process, has an ultrahigh value of 10% mass-loss temperature, T_d10_ of over 700 °C, and additionally excellent dielectric insulating properties, chemical stability, solvent resistance, and high mechanical strength. The T_d10_ of PBI was much higher than that of representative thermoresistant polymers [8].

Similar to commodity plastics, heat-resistant polymers have excellent electric insulating properties [9,10,11,12,13,14]. In other words, heat-resistant polymers such as PBI are electric insulators that can be used at high temperatures. However, the deterioration of electric insulating properties due to water absorption is quite serious for these polymers [15,16]. According to the literature, the electric resistivity of conventional PBI is distributed between approximately 10^7^ and 10^16^ Ω·cm depending on the humidity [9,10,11,13,14,17]. The conductivity of sulfopropylated PBI with water absorption of 27.6 wt% decreases from 10^−3^ S·cm^−1^ to 10^−5^ S·cm^−1^ with drying [18]. As can be seen from the wide distribution of electric resistivity or conductivity, the electric properties of PBI are significantly reduced by humidity, i.e., water absorption. Surprisingly, there are very few papers describing the correlation between electric conduction and water absorption in PBI.

In general, most polymers absorb atmospheric moisture, although their water absorption depends on the chemical structure. For example, the water absorption ratio of PBI is relatively high at 15–19% [19,20,21]. Other polymers with high water absorption include nylon 6 at 7.4% [22], aromatic polyimide at 2.51% [23], and epoxy resin CYCOM 977-2 at 3.4% [24]. Polymers with low water absorption include poly(ethylene terephthalate) at 1.1% [25] and poly(phenylene sulfide) at 0.026% [26]. Even for these polymers with low water absorption, the deterioration of electric properties due to water absorption is quite serious [27,28,29,30].

There are many studies published in the last century describing the reasons for the high water absorbency of PBI. Brooks et al. [21] reported that there are two possible interactions between water and PBI. One possibility is that two water molecules are bound to the imidazole ring, with one water molecule acting as a proton acceptor and the other as a proton donor. In this configuration, a maximum of four water molecules can be bound to each polymer repeating unit. A second possibility is the binding of one water molecule to two neighboring imidazole rings of adjacent polymer chains. In this case, the water molecule acts as both proton acceptor and proton donor. In this configuration, a maximum of two water molecules can be bound to each polymer repeating unit.

Thus, the water absorption process and its mechanism of PBI have been understood in detail. However, the causes of and remedies for the significant reduction in electric insulating properties of PBI due to water absorption remain unclear. To solve this problem, it is necessary to understand the formation of conductive paths and polarization mechanisms due to water absorption. In this study, we investigated the effect of water absorption on the electric conductivity and dielectric constant of ABPBI films, which show high water absorption and ultraheat-resistant property [9]. The effect of water absorption on the mobility of ABPBI chains was also investigated based on the spin–spin relaxation times obtained from NMR measurements.

## 2. Experimental Procedures

### 2.1. Polymer Syntheses

3,4-Diaminobenzoic acid (DABA) (purity: >97%) purchased from TCI (Tokyo Chemical Industry, Tokyo, Japan) was used as a monomer. Poly(phosphoric acid) (PPA, from TCI) was used as a polymerization solvent. Methanol, acetone, hexane, trifluoroacetic acid (TFA), and methanesulfonic acid (MSA) purchased from Kanto chemicals Co. Inc., Tokyo, Japan were used as solvents. Potassium hydroxide (KOH, from Kanto) and hydrochloric acid (HCl, from Kanto, Japan) were used as pH controllers. All the abovementioned reagents were used as received without further purification. PPA (30 g) was added to a three-necked round-bottom flask with a magnetic stirrer and heated at 100 °C for 1 h under a nitrogen atmosphere to remove moisture. Thereafter, DABA (730 mg, 4.8 mmol) was added to the flask to further remove the moisture by stirring at 100 °C for 1 h. After the monomer powders were completely dissolved in the reaction mixture, the mixture was heated and maintained at 180 °C for 6 h, 200 °C for 6 h, and 220 °C for 12 h. During this process, the reaction solution changed from red to dark brown and the viscosity increased significantly. The resulting mixture was dispersed in water to obtain a brown solid, which was ground into powder and thoroughly washed with water to remove PPA. Finally, 1 M NaOH was added dropwise to the dispersion with stirring until the pH reached 7.0. After filtration and drying under a vacuum, a dark yellow solid (808 mg) was obtained at 83% yield. The ABPBI (100.0 mg) film was cast onto a silicon substrate using TFA (2 mL) and one drop of MSA. After being dried at 25 °C, the film was scratched off the substrate and immersed in deionized water for 24 h to remove any residual solvents. The film was further dried at 80 °C for 12 h to obtain a self-standing film. The samples for water absorption and electric resistivity measurements were in a square shape with a size of 40 × 40 mm and a thickness of 56.8 µm.

### 2.2. Water Absorption Measurements

ABPBI films were dried in an oven at 150 °C under atmospheric pressure for 18 h. After drying, the films were placed in a container with saturated vapor pressure at room temperature for a certain period of time. The weight of the films was measured using an electric balance (A&D Co., Ltd., GH-200, Tokyo, Japan) before and after water absorption in a similar manner as described in previous studies [20,26]. The absorption ratio of the films, *Q*, was determined using the following equation:

(1) $$Q=\frac{W_{2}-W_{1}}{W_{1}}\times 100$$

Here, *W*_1_ and *W*_2_ are the weights of a film before and after water absorption, respectively. The volume fractions of water in the films after absorption, *ϕ*, was determined using the following equation:

(2) $$\phi =\frac{d_{p}\left(W_{2}-W_{1}\right)}{{d_{W}W}_{1}+d_{p}\left(W_{2}-W_{1}\right)}$$

Here, *d*_w_ and *d*_p_ represent the densities of pure water (= 1.00 g·cm^−3^) and ABPBI (= 1.30 g·cm^−3^), respectively.

### 2.3. Electric Resistance Measurements

The electric resistance of the films was measured at room temperature by the DC two-terminal method using a high-resistance meter (Hioki Electric Co., SM-7120, Ueda, Japan) and a parallel-plate electrode using electric guard (Hioki Electric Co., SME-8311, Ueda, Japan). The measurement using the parallel-plate electrode followed a general method for polymer films and satisfied the standard [31,32], which corresponds to the international standard [33]. The measurement was carried out in a chamber with controlled humidities (R.H. 9–21%). An electric potential of 10 V was applied in the direction of the film thickness, which corresponded to an electric field strength of 180 kV·m^−1^. The electric current in the present study ranged from 10^−11^ to 10^−5^ A, which was 10^5^–10^11^-fold higher than the maximum resolution of the high-resistance meter. The volume resistivity of the films, *ρ*_v_, was calculated using the following equation:

(3) $$R_{v}=\rho_{v}\frac{l}{S}$$

Here, *R*_v_ is the electrical resistance measured, *S* is the area of the electrode, and *l* is the thickness of the sample. In the present measurement, the integration time was set to 320 ms, which is the maximum value for the high-resistance meter. We measured the time profile of electric resistivity three times for the same sample. Then, the average values of electric resistivity from 150 to 180 s were determined, and the three resulting averages were used to determine the mean value and error.

### 2.4. Capacitance Measurements

The sample films were cut into a square shape with a size of 15 × 15 mm. The thickness of the films was 56.8 µm. The capacitance of the films was measured at 25.0 °C by the AC two-terminal method using an LCR meter (IM-3536, Hioki Electric Co., Ltd., Ueda, Japan) in a similar manner as described in previous studies [34,35]. The frequency range was set to 90 Hz to 8 MHz. Each film was sandwiched between brass electrodes of 10 mm in diameter, which were fabricated in the laboratory. An electric potential of 1.0 V was applied along the film thickness. The relative dielectric constant, *ε*_r_, of the films was calculated using the following equation:

(4) $$C=\varepsilon_{r}\varepsilon_{0}\frac{S}{l}$$

Here, *S* and *l* represent the area and thickness of the sample, respectively. *C* and *ε*_0_ are the electrostatic capacitance measured and the dielectric constant of vacuum (= 8.854 × 10^−12^ F·m^−1^), respectively.

### 2.5. NMR Measurements

The transverse relaxation (spin–spin relaxation) curves were measured by low-field nuclear magnetic resonance using an NMR spectrometer (MagnoMeter XRS™, Mageleka Inc., Kashiwa, Japan) at 17.951 MHz. The 180° pulse interval was typically 1000 µs with a 90° pulse length of 4.5 µs, and up to 20,000 echoes were recorded. The samples were dried at approximately 150 °C until just before they were set in test tubes for NMR measurements, which were 10 mm in diameter and 180 mm long. For the measurements of the samples after water absorption, the samples were allowed to absorb water using the same procedure as in the experiment of water absorption.

## 3. Results and Discussion

**Figure 1:**
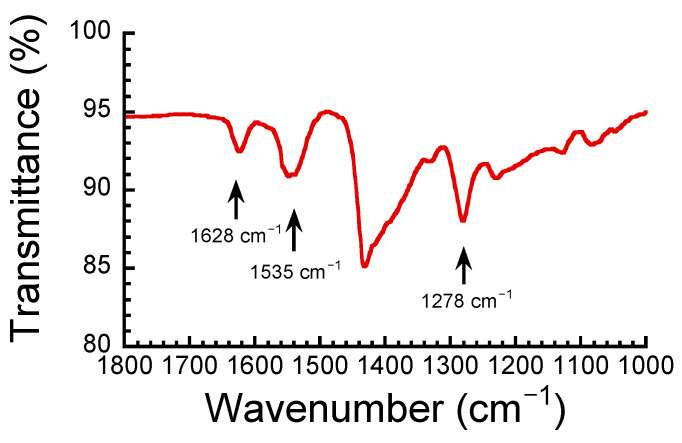
FTIR spectrum for the ABPBI films in a dry state used in the present study.

Figure 1 shows the IR spectrum for the ABPBI films in a dry state used in this study. The characteristic stretching vibrations of C=N, C=C, and C-N of the ABPBI films were assigned to be 1628, 1535, and 1278 cm^−1^, respectively. In addition, the broad absorption peaks that appeared at 3000–3500 cm^−1^ were attributed to N-H groups. Similar IR spectra were observed in past studies [36,37], indicating the successful synthesis of PBI.

**Figure 2:**
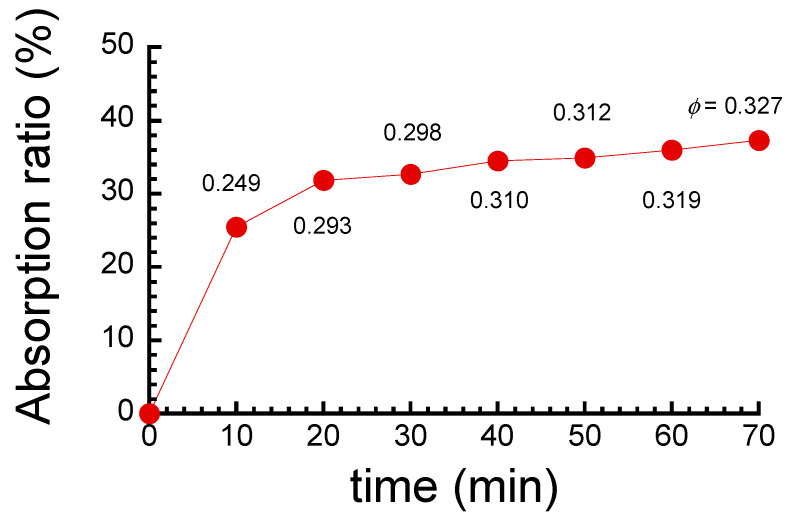
Relationship between water absorption ratio and absorption time of ABPBI films at room temperature.

Figure 2 shows the relationship between the water absorption ratio and absorption time of the ABPBI films. The absorption ratio gradually increased with time and became almost constant at 50 min. At equilibrium, the absorption ratio was 37%, which was higher than the values reported in the literature (= 15–19%) [19]. It was calculated that ~2.4 water molecules are bound to a unit of ABPBI at absorption equilibrium, which is close to the values reported by Brooks et al. [21]. As explained in the Introduction, the relatively high value of absorption ratio for PBI is considered to be due to the hydrogen bonding between benzimidazole imide ring and water molecules [38,39,40].

**Figure 3:**
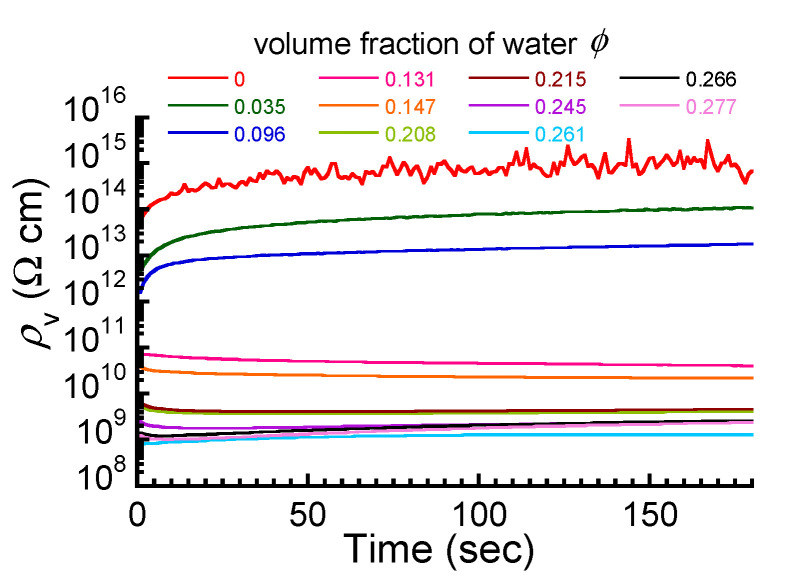
Time profiles of electric resistivity of ABPBI films at various volume fractions of water.

Figure 3 shows the time profiles of electric resistivity of the ABPBI films at various volume fractions of water. At volume fractions of *ϕ* < 0.096, the resistivity of the films gradually increased with time due to the absorbed current for charging [41]; then, it became almost constant at 120 s. At a volume fraction of *ϕ* = 0.147, the resistivity decreased slowly over time and became constant after approximately 140 s. At a volume fraction of *ϕ* = 0.277, the resistivity decreased in the first 10 s, then increased slowly, and became constant after 150 s. Thus, the resistivity of all films at various volume fractions was constant at 180 s, independently of the water absorption ratio. In this paper, the electric conductivity calculated from the equilibrium current observed at 180 s is discussed. Noise was seen in the resistivity at *ϕ* = 0, although no noise was seen in the resistivity of the films with water absorption. This is because the number of carriers of electric conduction increased due to water absorption, allowing sufficient current to flow for measurement. 

**Figure 4:**
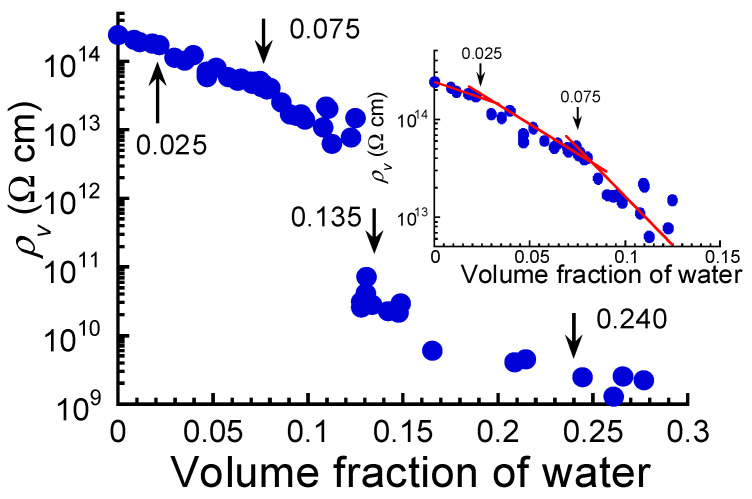
Electric resistivity at equilibrium as a function of the volume fraction of water for ABPBI films. The inset shows an enlarged graph of resistivity at volume fractions below 0.13. The arrows represent the volume fractions where electric conductivity transition occurs.

Figure 4 illustrates the electric resistivity at equilibrium as a function of the volume fraction of water for the ABPBI films. In a dry state (*ϕ* = 0), the resistivity of the films was 2.39 × 10^14^ Ω·cm, which was close to the value of 10^15^ Ω·cm reported by Ahmad et al. [17]. There have been many papers describing the resistivity of PBI films; however, the values vary widely across the studies, e.g., 10^12^–10^13^ Ω·cm in Aniruddha et al. [10], 10^−11^ S·cm^−1^ (10^11^ Ω·cm) in Ahmad et al. [11], and 10^17^ Ω·cm in Park et al. [13]. These low values of resistivity can probably be attributed to the fact that PBI and/or the measurement system contains water because of humidity. The resistivity of the films decreased with increasing volume fraction of water, and the slope of the line changed slightly at around *ϕ* = 0.025; then, it exhibited a clear change in slope at *ϕ* = 0.075, as shown in the inset of the figure. As the volume fraction increased further, the resistivity of the films exhibited a dramatic and discontinuous transition at a volume fraction of *ϕ* = 0.135. The resistivity of the films in a state of low resistivity was 1/82 of that in a state of high resistivity. After the transition, the resistivity of the films decreased gradually with the volume fraction and became almost constant at around *ϕ* = 0.240.

**Figure 5:**
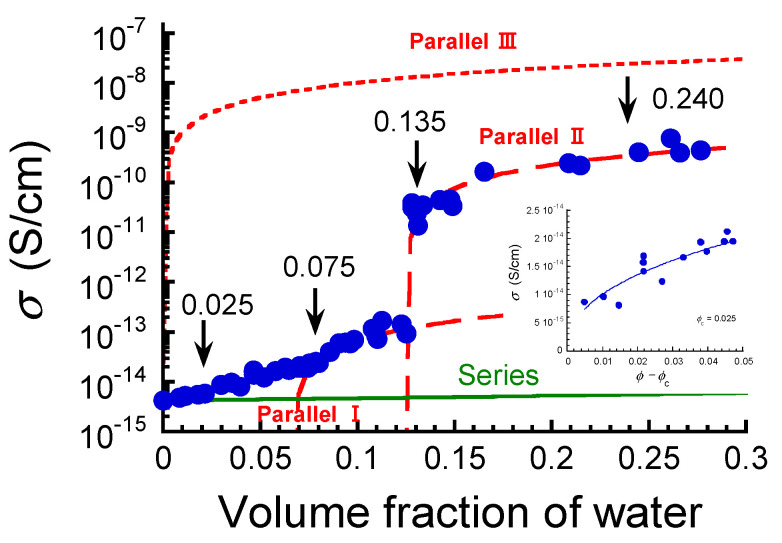
Relationship between electric conductivity and volume fraction of water for ABPBI films. Electric conductivity calculated by Equations (5) and (6) for series connection (solid line) and parallel connection (broken lines). The inset shows electric conductivity vs. *ϕ* − *ϕ*_c_. The arrows represent the volume fractions where electric conductivity transition occurs.

Figure 5 shows the relationship between the electric conductivity observed *σ*_obs_ (= *ρ*^−1^) and the volume fraction of water absorbed in the ABPBI films. The conductivity of the films can be explained by the following equations, which are obtained by modifying the basic equation [42], when two resistors made of pure water and ABPBI are connected in series and in parallel, respectively:

(5) $$\sigma_{\mathrm{obs}}=\frac{\sigma_{w}\sigma_{p}}{\phi \sigma_{p}+(1-\phi )\sigma_{w}}$$

(6) $$\sigma_{\mathrm{obs}}=\sigma_{p}\phi +\sigma_{w}(1-\phi )$$

Here, *σ*_p_ and *σ*_w_ are the electric conductivities of ABPBI and pure water, respectively. The experimental values of 4.2 × 10^−15^ and 10^−7^ S·cm^−1^ were substituted for *σ*_p_ and *σ*_w_, respectively. The solid line in the graph indicates the calculated values of conductivity obtained from Equation (5) for series connection. The experimental values of the films agreed with the calculated ones obtained from Equation (5) only at volume fractions of *ϕ* < 0.025. This strongly indicates that the electric current flows through both regions of ABPBI and water. In other words, this indicates that there is no conductive path flowing through either the region of water or that of ABPBI only.

At volume fractions of 0.025 < *ϕ*<0.075, the conductivity of the films satisfied the power law expressed by the following equation [43]:

*σ* = *σ*_0_(*ϕ* − *ϕ*_c_)^*β*^ (7)

where *σ*_0_ is the scaling factor, *ϕ*_c_ is the critical volume fraction, and *β* is the critical exponent related to the dimensionality of the percolated network of electric conduction. The inset shows the relationship between conductivity and *ϕ* − *ϕ*_c_ for the films at volume fractions above the critical volume fraction of *ϕ*_c_ = 0.025. The conductivity values showed a power dependency, as explained by Equation (7), suggesting that an increase in conductivity is considered to be a percolation behavior of electric conduction. Similar increase in conductivity (decrease in resistivity) as a result of water uptake was observed for an epoxy composite due to percolation conduction at the interfaces between the epoxy resin and fibers [15]. Thus, the shortest path of electric conduction with high conductivity occurred at *ϕ*_c_ = 0.025. The experimental value of the critical exponent was calculated to be 0.42, which coincided with the value for a three-dimensional lattice [44]. This strongly indicates that the electric conduction occurred three-dimensionally above the percolation threshold. Wang et al. [45] reported that a composite consisting of high-density polyethylene and natural fibers showed a significant increase in electric conductivity when the moisture content with respect to the maximum value reached approximately 50%. In our study, the water absorption ratio relative to the maximum value was calculated to be 41% when conductivity increased dramatically (at *ϕ* = 0.135). These values are almost identical; accordingly, serious deteriorations in the electric conductivity of polymer films occur approximately in this region of absorption ratio.

At volume fractions of 0.075 < *ϕ* < 0.135, the conductivity of the films was much higher than the value calculated by Equation (7), indicating that multiple conduction paths bridging between the electrodes occurred. Actually, the conductivity in this region could be well represented by Equation (6) using the values of *σ*_p_ = 2 × 10^−14^ and *σ*_I_ = 2 × 10^−12^ (Parallel I). *σ*_I_ represents the conductivity of a domain of ABPBI that absorbs water, which is the maximum conductivity achieved in this region. This maximum value might be dominated by the spatial distribution of water in the film. At a volume fraction of 0.135, the conductivity of the films increased dramatically. Interestingly, the conductivity at volume fractions of *ϕ* ≧ 0.135 could also be well represented by Equation (6) using the values of *σ*_p_ = 1.2 × 10^−13^ and *σ*_II_ = 3 × 10^−9^ (Parallel II). *Σ*_II_ represents the conductivity of a domain of ABPBI that absorbs more water than the region of Parallel I, which is also the maximum conductivity achieved in this region. It should be emphasized here that the observed conductivity can be decomposed into the components explained by Parallel I and Parallel II. The physical meaning of the conductivity maxima in each of these regions requires further investigation. At higher volume fractions, the experimental values approached the calculated values of parallel connection (Parallel III), but the experimental value at a volume fraction of 0.276 was 1/64 of the calculated one.

**Figure 6:**
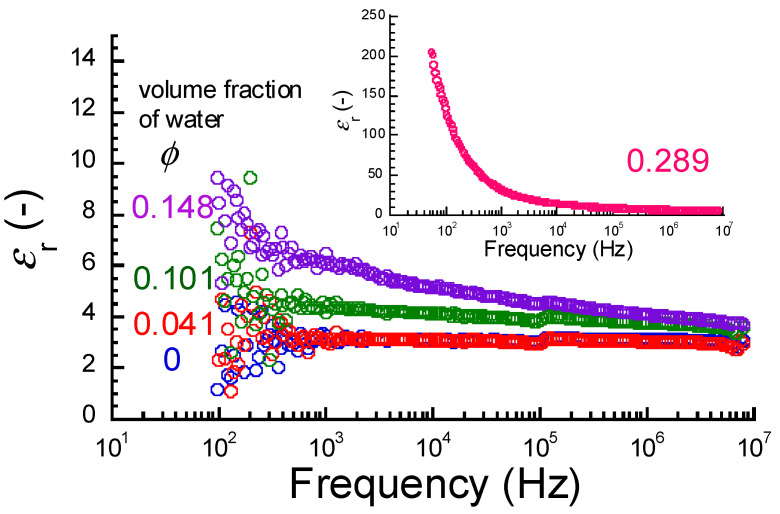
Frequency spectra of relative dielectric constant of ABPBI films at various volume fractions of water (inset: *ϕ* = 0.289).

Figure 6 exhibits the frequency spectra of the relative dielectric constant of the ABPBI films at various volume fractions of water. The dielectric constant of the films at *ϕ* = 0 and 0.101 was independent of the frequency for the whole region. At volume fractions of *ϕ* > 0.101, the dielectric constant at low frequencies showed a high value and decreased gradually with increasing frequency. Similar behavior of dielectric constant was seen in polyimide films with water sorption [23]. No dielectric relaxation or dielectric dispersion occurs for ABPBI and pure water in this frequency range. Therefore, the observed high dielectric constant at low frequencies is considered to be macroscopic dielectric polarization due to mobile ions traveling between the electrodes. The water absorption regions 

**Figure 7:**
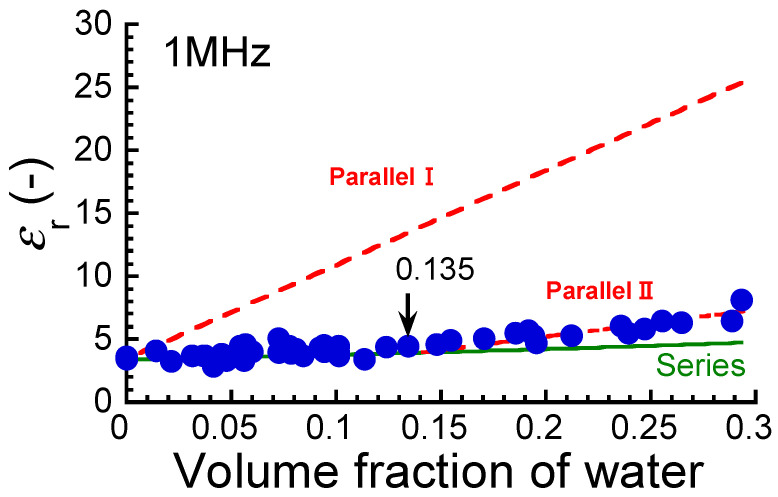
Relationship between relative dielectric constant at 1 MHz and the volume fraction of water for ABPBI films. Dielectric constant calculated by Equations (8) and (9) for series connection (solid line) and parallel connection (broken lines). The arrows represent the volume fractions where electric conductivity transition occurs.

Figure 7 shows the relationship between the relative dielectric constant at 1 MHz and the volume fraction of water for the ABPBI films. The dielectric constant of a dried film was measured to be 3.4. Aniruddha et al. [10] measured the dielectric constant of ABPBI films at room temperature using the same LCR meter used in this study. The dielectric constant was reported to be 3.4. Ahmad et al. [11] measured the dielectric constant of ABPBI films using an Alpha-ATB high-resolution dielectric analyzer and reported it to be 6.18. Han et al. [12] measured the dielectric constant of PBI films using an impedance analyzer and reported it to be 4. When two capacitors made of pure water and polymer are connected in series, the observed dielectric constant of the films, *ε*_obs_, is expressed by the following equation, which is obtained by modifying the basic equation [42]:

(8) $$\varepsilon_{obs}=\frac{\varepsilon_{w}\varepsilon_{p}}{\phi \varepsilon_{p}+(1-\phi )\varepsilon_{w}}$$

Here, *ε*_w_ and *ε*_p_ are the dielectric constants of pure water and polymer, respectively. *ϕ* is the volume fraction of pure water. When two capacitors are connected in parallel, the observed dielectric constant of the films, *ε*_obs_, is expressed by the following equation:

(9) $$\varepsilon_{obs}=\phi \varepsilon_{w}+\left(1-\phi \right)\varepsilon_{p}$$

The notations of the symbols are the same as those in Equation (8). The solid (Series) and broken (Parallel I) lines in the graphs represent the dielectric constants calculated for series and parallel connections, respectively, substituting with the values of *ε*_w_ = 78.3 and *ε*_p_ = 3.4. The experimental values agreed with the calculated ones obtained from Equation (8) only at volume fractions of *ϕ* < 0.135. This strongly indicates that the absorbed water is not uniformly distributed in the films. No obvious change in the dielectric constant was observed at *ϕ* = 0.075. At volume fractions of *ϕ* ≧ 0.135, the experimental values were clearly higher than the calculated ones obtained from Equation (8). This could be attributed to the effect of the capacitors connected in parallel. Furthermore, the increase in dielectric constant at volume fractions of *ϕ* > 0.135 was not large, which was completely different from the behavior seen for electric conductivity. The broken line in the figure represents the dielectric constant calculated by Equation (8), substituting with the values of *ε*_w_ = 25 and *ε*_p_ = 3.8 (Parallel II). The experimental values were in good agreement with the calculated ones, suggesting that a parallel connection occurred at *ϕ* = 0.135.

**Figure 8:**
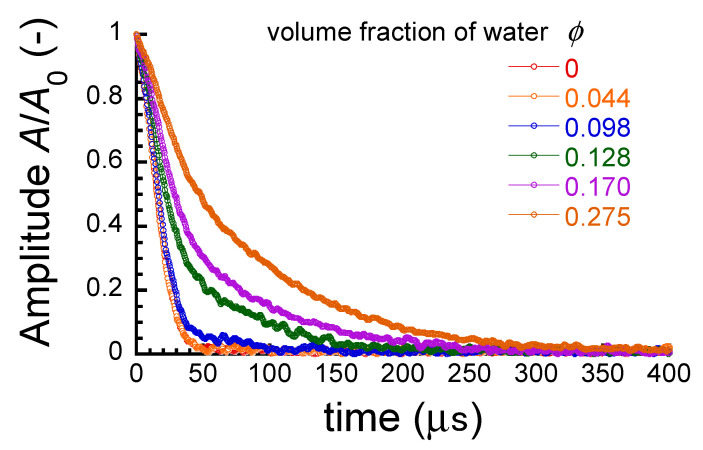
*T*_2_ relaxation curves for ABPBI films at various water absorption ratios.

Figure 8 shows the relaxation curves of signal intensity *A*/*A*_0_ for the ABPBI films at various volume fractions of water. *A*/*A*_0_ represents the signal intensity at a certain time with respect to that at *t* = 0. The observed relaxation curves for the films were fitted with a double-exponential decay as follows [46]:

(10) $${A/A}_{0}=A_{1}\exp\left(-{\left(\frac{t}{\tau_{1}}\right)}^{2}\right){+A}_{2}\exp\left(-\frac{t}{\tau_{2}}\right)+A_{\infty }$$

where *A*_1_ and *A*_2_ represent the intensity ratio for fast and slow relaxations, respectively. *τ*_1_ and *τ*_2_ represent the relaxation time for fast and slow relaxations, respectively. *A*_∞_ is a constant that is independent of time. The results of fitting using Equation (10) are shown in Table 1. As shown in the correlation coefficient *r*, the values were higher than 0.99668 at all volume fractions, indicating that the observed relaxation could be well explained by Equation (10), which consists of two relaxation processes.

**Figure 9:**
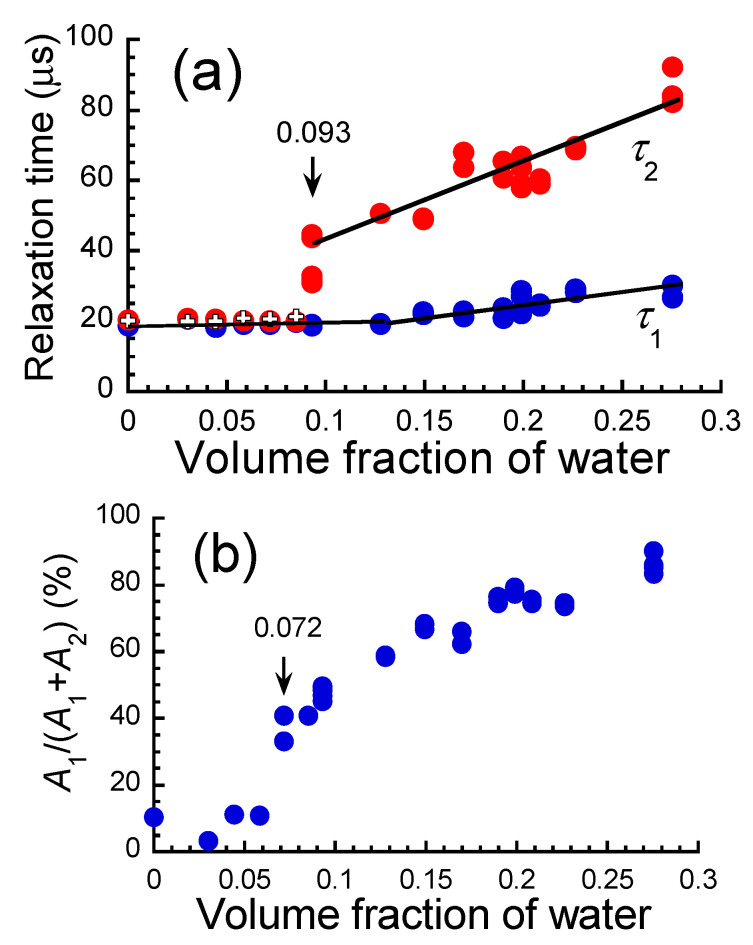
(**a**) Relaxation times *τ*_1_ and *τ*_2_ and (**b**) intensity ratio of *A*_2_/(*A*_1_ + *A*_2_) for ABPBI films as a function of the volume fraction of water. White crosses in Figure 9a represent the relaxation times obtained by fitting with only the first term in Equation (10). The arrows represent the volume fraction at where abrupt change occurs.

Figure 9a exhibits the relationship between the relaxation times τ_1_ and τ_2_ in Equation (10) and the volume fraction of absorbed water for the ABPBI films. At volume fractions of *ϕ* < 0.093, *τ*_1_ and *τ*_2_ had almost the same values (*τ*_1_~19 µs, *τ*_2_~20 µs), which were equal to the relaxation time for the films without water absorption, and the relaxation times were independent of the volume fraction of water. Reuvers et al. [47] performed a beautiful work involving NMR imaging and relaxation studies of water-absorbed nylon 6 and found peaks at around 0.6 and 0.07 ms, and they reported that these are caused by the relaxation of amorphous regions of nylon 6 with high and low mobilities, respectively. The relaxation time for bulk water is 1.8 ms, which is much longer than these relaxation times. In other words, it is reasonable to assume that the relaxation seen in these timescales is due to the mobility of the polymer chains, and not the pool of ^1^H nuclei of water absorbed. The relaxation times τ_1_ and τ_2_ observed for the ABPBI films in this study are on the same timescale as the relaxation time for the amorphous low-mobility region of nylon 6. Therefore, *τ*_1_ and *τ*_2_ are considered to reflect the mobility of ABPBI chains in a region of low mobility. In this region of volume fractions, the relaxation time of ABPBI is independent of the volume fraction. This suggests that the relaxation time of ABPBI is not affected by the chain mobility of ABPBI, although ABPBI absorbs water. At volume fractions of *ϕ* ≧ 0.130, *τ*_1_ increased slightly with the volume fraction. In contrast, *τ*_2_ increased abruptly and markedly at *ϕ* = 0.10. This suggests that the slow relaxation is due to the mobility of ABPBI chains, which is significantly affected by water absorption. *τ*_2_ increased proportionally with the volume fraction with a steep slope. This suggests that the mobility of ABPBI chains increases as the volume fraction of water increases, i.e., as water absorption proceeds. The results of fitting the relaxation curve for the intensity ratio *A*/*A*_0_ with only the first term in Equation (10) are also shown in the figure. The relaxation times obtained were the same as the results for *τ*_1_ and *τ*_2_ fitted with Equation (10). Therefore, the relaxation curve for the intensity ratio *A*/*A*_0_ should be fitted with a single relaxation at volume fractions of *ϕ* < 0.093. As mentioned above, this suggests that the mobility of PBI chains is not affected by the absorbed water in this region of volume fractions, although ABPBI absorbs water.

Figure 9b shows the relationship between the intensity ratio of *A*_2_/(*A*_1_ + *A*_2_) in Equation (10) and the volume fraction of water for the ABPBI films. The intensity ratio was constant at approximately 5% at volume fractions of *ϕ* < 0.072. The intensity ratio suddenly and dramatically increased at *ϕ* = 0.072. This volume fraction was slightly lower than the volume fraction at which *τ*_2_ increased dramatically. In addition, this volume fraction was almost identical to the volume fraction whose conductivity and dielectric constant can be described by a model of parallel connection. Thus, it can be considered that the mechanism of the sudden increase in the intensity ratio is similar to that of the sudden increase in conductivity and dielectric constant. At a volume fraction of *ϕ* = 0.275, the intensity ratios of *A*_1_ and *A*_2_ were 14% and 86%, respectively.

**Figure 10:**
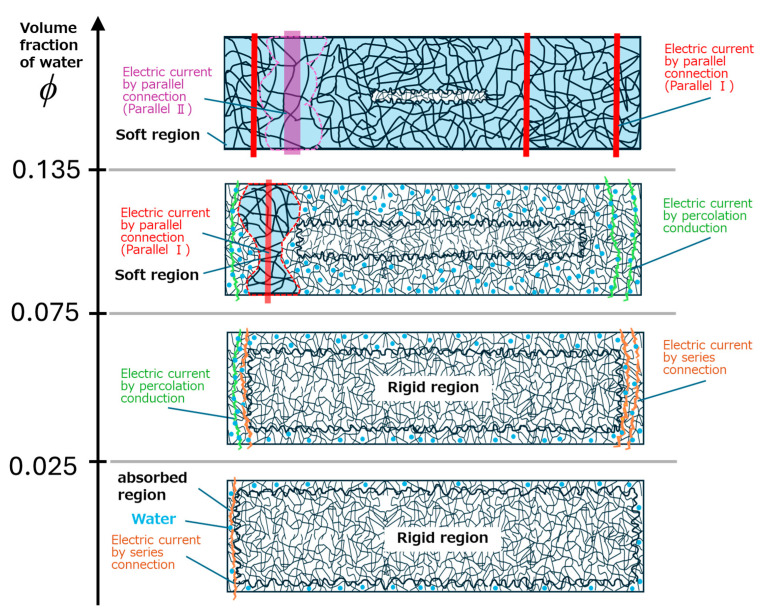
Schematic illustrations representing the mechanism underlying the significant deterioration in electric properties of ABPBI films due to water absorption.

Figure 10 displays schematic illustrations explaining the dramatic transition in electric resistivity for ABPBI films due to water absorption. At volume fractions of *ϕ* < 0.025, it is considered that electric conduction occurs near the edges of the film. It is assumed that water infiltrates the same distance from all surfaces of the film and that the probability of water presence is high near the edges. Electric conduction is considered to occur through both ABPBI and water microscopically and is uniformly dispersed in the film. It is because the measured conductivity follows a model of series connection of water and ABPBI. At volume fractions of 0.025 ≦ *ϕ* < 0.075, electric conductivity follows the percolation theory. This indicates that a three-dimensional path of electric conduction is formed at *ϕ* = 0.025, and the path develops in three dimensions as water absorption increases. Of course, the conductive path of the series connection at *ϕ* < 0.025 does not disappear even at *ϕ* > 0.025, although its contribution to the total current is low. The T_2_ relaxation for a wet ABPBI film at volume fractions of *ϕ* < 0.075 is single and the relaxation times are equal to that of a dried one. This means that the water absorbed is microscopically dispersed in the ABPBI film and the mobility of ABPBI chains is not altered by water absorption, i.e., ABPBI remains in a glassy state. At a volume fraction of 0.075, a conductive path with high electric conductivity is produced due to water absorption, in addition to the path of percolation conduction at *ϕ* < 0.075. At this volume fraction, the NMR relaxation curves split into two relaxation processes of fast and slow relaxation, suggesting a region of low chain mobility (a softened region) is formed in the film. These results evidently indicate that the formation of the softened region relates to high conductivity. Accordingly, it is considered that the high-conductivity path is not a region of just water but a softened region of PBI that absorbs water. At volume fractions of 0.075 ≦ *ϕ* < 0.135, the softened region with high conductivity is expanded with increasing volume fraction of water. At a volume fraction of 0.135, a conductive path with significantly higher electric conductivity appears due to water absorption. At this volume fraction, there are no anomalies in both NMR relaxation intensity and relaxation time, and these values increase monotonically with increasing volume fraction. It is reasonable to assume that the softened region is further softened as the volume fraction is increased. Therefore, a conductive path with high conductivity is presumed to appear when the softness of the softened region has reached a certain value. These two transitions of electric conductivity observed in this study may be due to the increase in ion mobility by changing the mesh size of entangled ABPBI chains.

## 4. Conclusions

The effect of water absorption on the electric properties of ABPBI films was investigated to reveal the degradation of electric insulating properties of the films. The volume fraction at which the largest increase in conductivity (*ϕ* = 0.135) was observed was nearly equal to the volume fraction at which the longest relaxation time appeared. This suggests that conductive ions are transported mainly via softened regions that have acquired high mobility of polymer chains via water absorption. This means, conversely, if the chain mobility can be suppressed, significant deterioration of the insulating properties can be prevented. On the other hand, the dielectric constant increased slightly in proportion to the volume fraction of water and did not exhibit a significant increase, suggesting that the low dielectric properties of ABPBI do not disappear with water absorption. To suppress the degradation of electric insulating properties of ABPBI films due to water absorption, it is necessary to prevent the creation of softened regions, or even if these regions are created, it is necessary to control the spatial distribution of these regions. An analysis of the main ν(NH) band using FTIR and density functional theory calculations [48] would be a very powerful tool in elucidating the mechanism underlying the creation of softened regions due to water absorption.

## Figures and Tables

**Table 1:** The results of fitting with Equation (10) of the NMR spectra for ABPBI films at various volume fractions of water.

	Short		Long		
*ϕ* ^a^	*A*_1_/(*A*_1_ + *A*_2_) (%) ^b^	*τ*_1_ (μs) ^c^	*A*_1_/(*A*_1_ + *A*_2_) (%) ^d^	*τ*_2_ (μs) ^e^	*r* ^f^
0	89.6	18.8	10.4	20.3	0.99818
0.044	88.8	18.4	11.2	20.6	0.99668
0.098	55.3	19.5	44.7	23.0	0.99901
0.128	41.6	19.4	58.4	50.7	0.99920
0.170	37.8	22.9	62.2	68.1	0.99935
0.275	16.6	26.9	83.4	92.2	0.99963

^a^ volume fraction of water, ^b^ Intensity ratio of fast process, ^c^ relaxation time of fast process, ^d^ Intensity ratio of slow process, ^e^ relaxation time of slow process, ^f^ correlation coefficient.

## Data Availability

Data are contained within the article.
